# Increased Incidence of Legionellosis after Improved Diagnostic Methods, New Zealand, 2000–2020

**DOI:** 10.3201/eid2906.221598

**Published:** 2023-06

**Authors:** Frances F. Graham, David Harte, Jane Zhang, Caroline Fyfe, Michael.G. Baker

**Affiliations:** University of Otago, Wellington, New Zealand (F.F. Graham, J. Zhang, C. Fyle, M.G. Baker);; Environmental Science and Research, Wellington (D. Harte)

**Keywords:** epidemiology, increased incidence, improved diagnostic methods, Legionnaires’ disease, legionellosis, Legionella, bacteria, New Zealand

## Abstract

Legionellosis, notably Legionnaires’ disease, is recognized globally and in New Zealand (Aotearoa) as a major cause of community-acquired pneumonia. We analyzed the temporal, geographic, and demographic epidemiology and microbiology of Legionnaires’ disease in New Zealand by using notification and laboratory-based surveillance data for 2000‒2020. We used Poisson regression models to estimate incidence rate ratios and 95% CIs to compare demographic and organism trends over 2 time periods (2000–2009 and 2010–2020). The mean annual incidence rate increased from 1.6 cases/100,000 population for 2000–2009 to 3.9 cases/100,000 population for 2010–2020. This increase corresponded with a change in diagnostic testing from predominantly serology with some culture to almost entirely molecular methods using PCR. There was also a marked shift in the identified dominant causative organism, from *Legionella pneumophila* to *L. longbeachae*. Surveillance for legionellosis could be further enhanced by greater use of molecular typing of isolates.

Legionellosis is caused by the gram-negative bacterium *Legionella*. This infection is predominantly the consequence of an environmental exposure to legionellae, which are ubiquitous in water and moist soil ecosystems. Incidence and seroprevalence studies show that the infection has a global distribution (*1*,*2*). The severity of disease varies from mild febrile illness (Pontiac fever, incubation period commonly 24‒48 hours) (*3*) to serious and sometimes fatal pneumonia (Legionnaires’ disease, incubation period commonly 2‒10 days) (*4*). Recognized infection risk factors for legionellosis include smoking, chronic obstructive pulmonary disease, diabetes, various conditions associated with immunodeficiency, male sex, and increasing age (*4*).

Disease surveillance for legionellosis began in New Zealand (Aotearoa) in 1979 with the collection of laboratory-based data (cases positive for *Legionella* species); additional data were available starting in June 1980, when the disease became notifiable (*5*). A review of national surveillance data for 1979‒2009 showed that the annual incidence rate for laboratory-identified cases was 2.5 cases/100,000 persons and 1.4 cases/100,000 persons for notified cases (*6*); the disparity was caused by not all laboratory-identified cases being notified.

Inhalation of aerosolized bacteria from an environmental source is the usual means of *Legionella* transmission. Environmental surveillance shows that *Legionella* species are widely distributed in New Zealand (*7*). Commonly identified sources are engineered environments, such as wet cooling towers and water distribution systems, which can be reservoirs and amplifiers of the bacteria, particularly *L. pneumophila* (*1*). Aerosolized dust inhalation from handling compost and potting mix materials is most probably a major transmission route contributing to the cases of legionellosis caused by *L. longbeachae* (*8*), which is the predominant species that causes disease in New Zealand (*9*).

During 1980‒2000, all cases of legionellosis were diagnosed in New Zealand by using traditional laboratory methods, notably culture isolation and direct fluorescent-antibody staining of respiratory tract specimens, and serology by immunofluorescent antibody testing. During that period, the *Legionella* urinary antigen test (UAT), followed by nucleic acid amplification test (NAAT), became established diagnostic tools. Since 2000, there has been an increasing shift toward NAATs, primarily PCR testing, and this method has dominated from 2015 onward.

The aims of this study were to provide an updated analysis of the epidemiology of legionellosis in New Zealand, focusing on differences between 2 periods (2000–2009 and 2010*–*2020); examine the influence of changing diagnostic methods on the temporal and geographic distribution of notified and laboratory-identified legionellosis cases; and review changes in the causative species. Our findings are intended to be used to improve surveillance, prevention, and management of legionellosis.

## Methods

### Surveillance Data and Case Definitions

We used 2 data sources to describe the epidemiology of legionellosis (including Legionnaires’ disease and Pontiac fever) in New Zealand: notifiable disease data and laboratory-based surveillance data (Appendix). We analyzed all reported cases of Legionnaires’ disease and Pontiac fever. Pontiac fever cases are a small proportion of legionellosis notifications (3 cases, 0.1% during 2000‒2020), possibly because Pontiac fever is a milder and self-resolving illness, which consequently is mostly untested and therefore unreported (*6*).

A confirmed case of legionellosis requires a clinically compatible disease with >1 form of laboratory evidence: *Legionella* culture isolated from a clinical specimen; a >4-fold increase in immunofluorescent antibody titer against *Legionella* spp. to >256 between acute-phase and convalescent-phase paired serum samples tested in parallel by using pooled antigen at the same reference laboratory; and detection of *L*. *pneumophila* serogroup 1 by antigen in urine or positive NAAT result (*10*). A probable case is defined as a clinically compatible disease with laboratory evidence of *Legionella* infection showing >1 antibody titers >512 but without a demonstrated 4-fold titer increase.

### Statistical Analysis

We used Poisson regression to estimate the incidence rates, age-standardized to 2013 New Zealand census population age-structure, for legionellosis over 2 periods: 2000*–*2009 (preceding a major shift to NAAT) and 2010*–*2020 (after the effect of a shift to NAAT and change in diagnostic criteria/case definition). The null hypothesis was that there was no change in the rates of legionellosis cases in these time periods. We calculated the annual incidence rate (legionellosis cases/100,000 population) by dividing reported cases by each mid-year census estimate and multiplying by 100,000. We did not calculate rates when a category had <5 notified cases. We performed statistical analysis by using SAS version 9.4 (SAS Institute, https://www.sas.com). 

## Results

### Case Incidence and Temporal Trends

**Figure 1:**
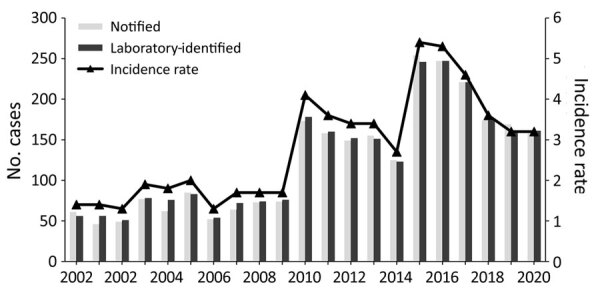
Notification and laboratory-identified case numbers and incidence rates (cases/100,000 population), by year, in study of increased incidence of legionellosis after improved diagnostic methods, New Zealand, 2000–2020.

A total of 2,628 legionellosis cases were notified during 2000‒2020, an overall mean annual incidence rate of 2.7 cases/100,000 population. The mean annual incidence rate increased from 1.6 cases/100,000 population in 2000–2009 to 3.9 cases/100,000 population in 2010–2020 (Figure 1). We observed marked increases in legionellosis cases in 2003, 2010, and 2015 and decreases in the periods between those years (Appendix Table 1).

A total of 2,675 laboratory-identified cases that fit the case definition were reported during the study period. Of the laboratory-identified cases that met the case definition, 1,942 (72.6%) were confirmed and 733 (27.4%) were probable. The incidence rate for laboratory-identified cases (confirmed and probable cases combined) averaged 2.8 cases/100,000 population/year (range 1.3 cases/100,000 population in 2002 and 2006 to 5.4 cases/100,000 population in 2015) (Figure 1).

### Causative *Legionella* Species

**Figure 2:**
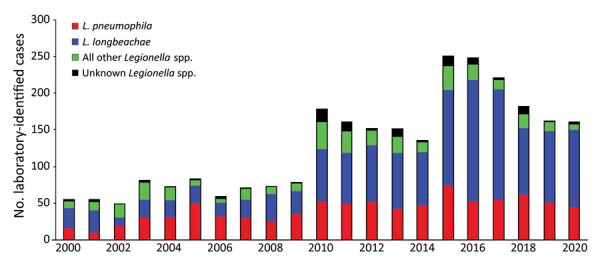
Laboratory-identified legionellosis cases, by species and year, in study of increased incidence of legionellosis after improved diagnostic methods, New Zealand, 2000–2020.

We grouped the number of *Legionella* species identified through laboratory-based surveillance during 2000‒2020 as *L. longbeachae*, *L. pneumophila*, or other (Figure 2; Appendix Table 2). *L. longbeachae* was identified as the causative agent for 51.0% of all legionellosis cases over the 21-year period (Appendix Table 2). *L. pneumophila* accounted for 31.2% of all cases, followed by other *Legionella* spp. (13.5%) and unidentified *Legionella* spp. (4.3%) (Appendix Table 2).

During 2000‒2009, the annual laboratory-identified clinical case numbers caused by *L. pneumophila* infection were similar to those caused by *L. longbeachae* (*L. pneumophila*, 26.4 cases/year; *L. longbeachae*, 25.2 cases/year). During 2010‒2020, *L. longbeachae* case numbers increased 4-fold to average 101.3 cases/year (55.6%, 1,114/2,002 cases), compared with a doubling in *L. pneumophila* case numbers to an average of 51.8 cases/year (28.5%, 571/20,02 cases). We found a marked increase in the number of *L. longbeachae* cases in which the serogroup was unidentified (32 cases in 2010 increasing to 61 cases in 2020); those cases were identified by using a molecular method that did not differentiate between *L. longbeachae* serogroups 1 and 2. (Appendix Table 2).

### Method of Case Identification

**Figure 3:**
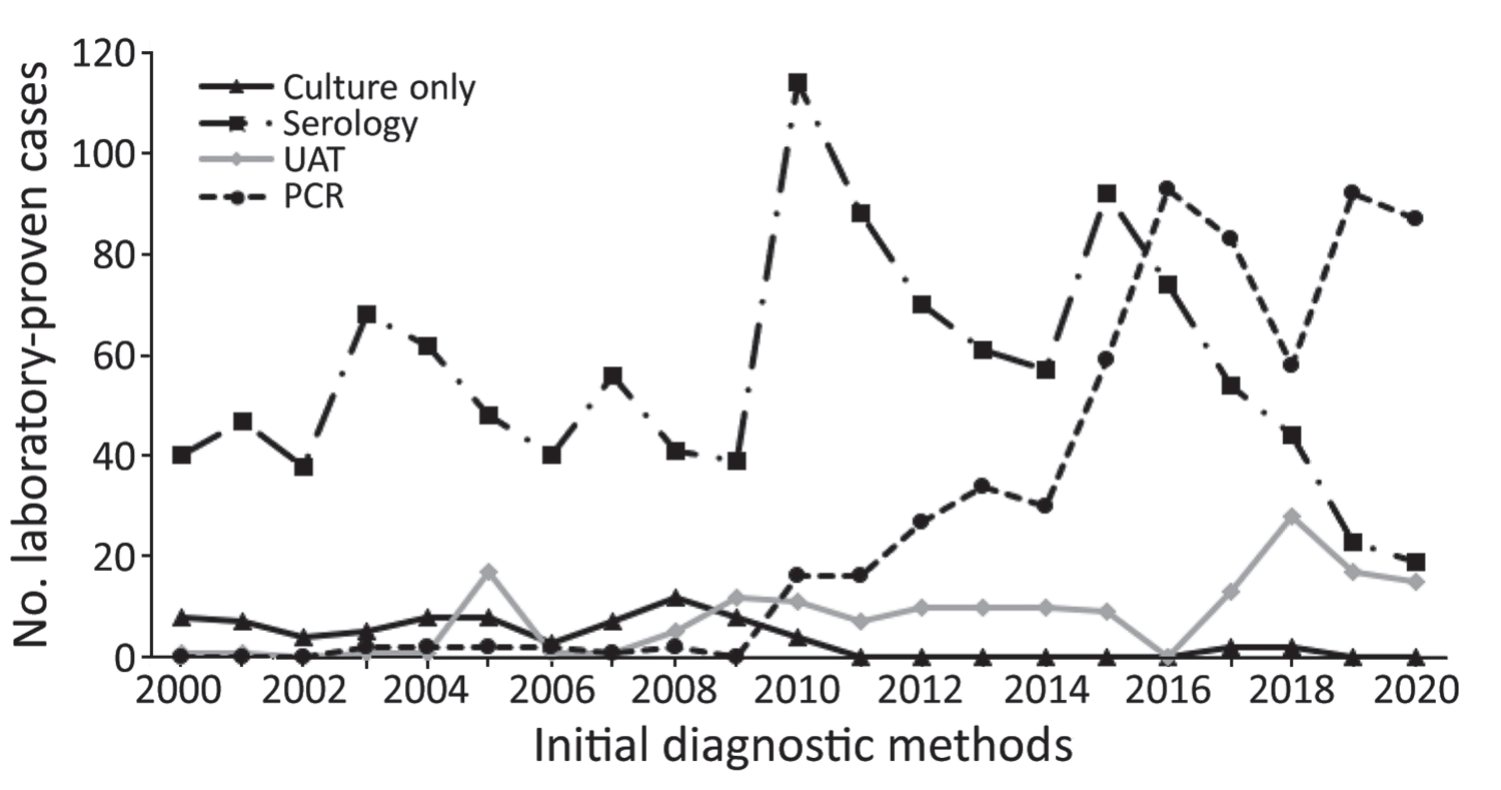
Laboratory-identified legionellosis, by initial diagnostic method and year, in study of increased incidence of legionellosis after improved diagnostic methods, New Zealand, 2000–2020. UAT, urine antigen test.

The method of initial diagnosis that gave a positive *Legionella* result changed over time (Figure 3; Appendix Table 3). The number of cases diagnosed by using PCR increased progressively from 2010 onward, whereas the number of cases diagnosed by traditional methods of serology and culture isolation alone decreased. This observation is reinforced (Appendix Table 3) and shows a major increase in cases diagnosed by using molecular methods between the 2 10-year periods compared with more traditional methods (culture, serology, and UAT).

### Demographic Characteristics

**Figure 4:**
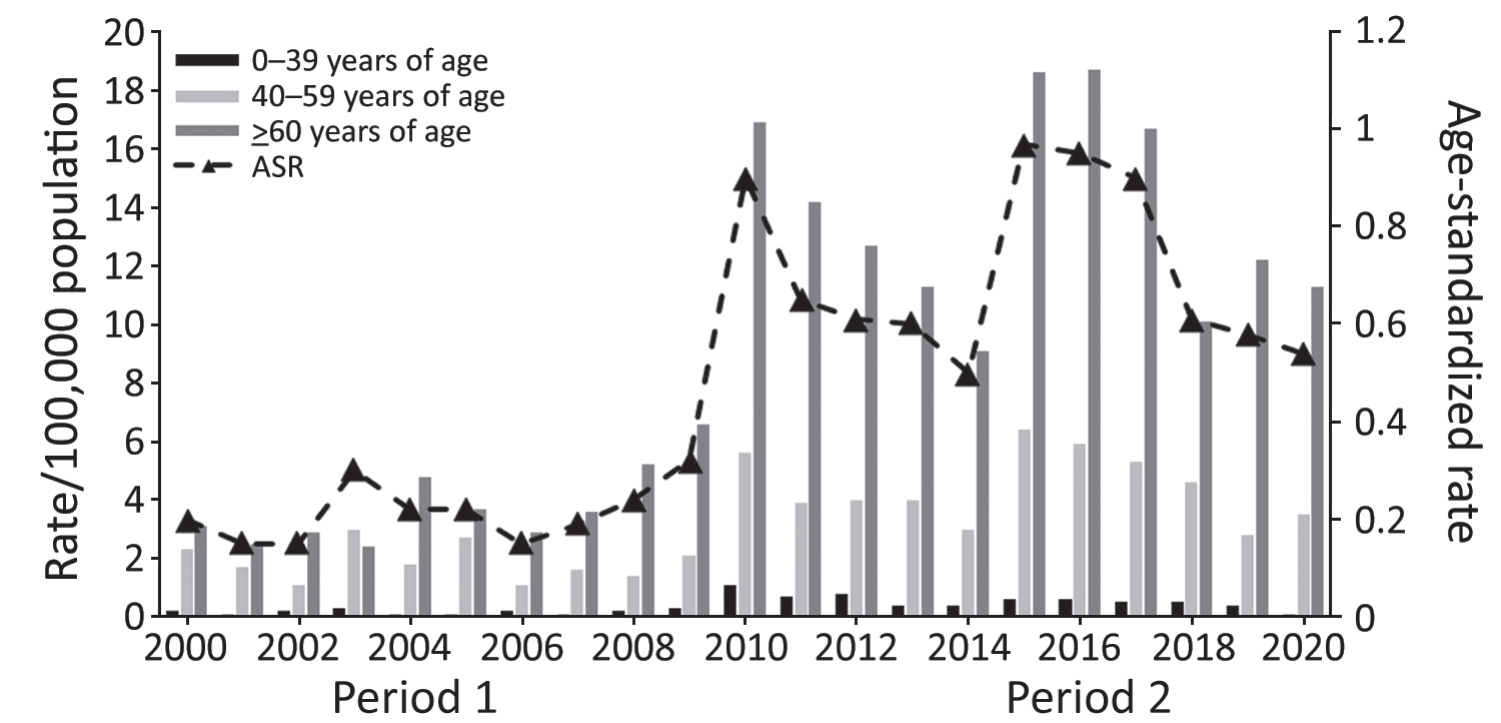
Incidence rate and ASR of legionellosis notifications, by age group and year (time-period), in study of increased incidence of legionellosis after improved diagnostic methods, New Zealand, 2000–2020. Period 1, 2000–2009; period 2, 2010–2020.ASR, age-standardized rate.

Except for 2003, legionellosis incidence and age-standardized rates were highest in adults >60 years of age, followed by adults 40‒59 years of age and children and younger adults (0‒39 years of age) (Table 1; Figure 4). Compared with 2000–2009, legionellosis incidence and age-standardized rates were much higher in 2010–2020. Rates increased across all age, sex, and ethnic groups (Table 1; Appendix Tables 4, 5). The association of age with the incidence of legionellosis demonstrates increasing incidence with age for all *Legionella* species, especially in the population >60 years of age (Table 1). During 2000*–*2020, the number of notified cases (n = 2,628) was also higher for male (62.7%) than female (37.1%) patients, an overall ratio of 1.7:1.

**Table 1:** Characteristics of notified case-patients by age group, sex, ethnicity, and *Legionella* species, New Zealand, 2000–2020*

Category	2000–2009					2010–2020					Comparison of 2 periods, IRR (95% CI)
	No. cases	Crude rate	ASR† (95% CI)	IRR (95% CI)		No. cases	Crude rate	ASR† (95% CI)	IRR (95% CI)		
Age group, y											
Total	643	1.6	1.5 (1.4–1.6)	NA		1,985	3.9	3.8 (3.6–3.9)	NA		2.5 (2.3–2.7)
<1–39	46	0.2	NA	Referent		99	0.4	NA	Referent		1.7 (1.2–2.9)
40–59	219	1.9	NA	9.1 (6.6–12.5)		619	5.0	NA	12.8 (10.3–15.8)		2.5 (2.1–2.9)
>60	378	4.5	NA	21.9 (16.1–29.7)		1,267	11.8	NA	32.8 (26.7–40.2)		2.6 (2.3–2.9)
Sex											
M	383	1.9	1.9 (1.7–2.11)	1.7 (1.4–2.0)		1,265	5.0	5.0 (4.7–5.2)	1.9 (1.8–2.1)		2.6 (2.3–2. 9)
F	254	1.2	1.1 (1.0–1.3)	Referent		720	2.7	3.0 (2.4–2.8)	Referent		2.3 (2.0–2.6)
Unknown	6	NA				0	NA				
Ethnicity											
European	517	2.3	2.1 (1.9–2.3)	Referent		1,620	4.5	3.9 (3.7–4.1)	Referent		1.85 (1.67–2.04)
Māori	27	0.5	0.7 (0.4–1.1)	0.35 (0.22–0.55)		149	1.7	2.8 (1.4–3.3)	0.73 (0.61–0.88)		3.89 (2.42–6.25)
Pacific Peoples	13	0.6	0.7 (0.3–1.1)	0.32 (0.17–0.58)		72	1.7	3.1 (2.3–3.9)	0.80 (0.62–1.03)		4.63 (2.42–8.88)
Other	17	0.2	0.2 (0.1–0.4)	0.11 (0.07–0.19)		93	1.1	1.7 (1.4–2.1)	0.45 (0.36–0.56)		7.46 (4.31–12.9)
Unknown	69	NA				51	NA				
Laboratory status											
Confirmed	537	1.3	0.9 (0.8–0.9)	Referent		1,700	3.3	2.4 (2.2–2.5)	Referent		2.7 (2.5–3.0)
Probable	106	0.2	0.2 (0.1–0.2)	0.2 (0.2–0.23)		285	0.6	0.4 (0.3–0.4)	0.2 (0.1–0.2)		2.2 (1.7–2.7)
*L. pneumophila*											
Total	256	6.2	NA	NA		527	11.2	NA	NA		1.80 (1.57–1.94)
<1–39	9	0.3		Referent		58	0.8		Referent		2.67 (2.31–2.86)
40–59	58	1.0		1.41 (0.97–1.73)		196	2.9		2.85 (2.19–3.12)		2.90 (2.50–3.04)
>60	189	3.2		13.6 (9.42–16.5)		273	4.9		5.15 (3.99–5.63)		1.53 (1.33–1.88)
*L. longbeachae*											
Total	161	3.9	NA	NA		1,062	22.6	NA	NA		5.79 (5.49–6.12)
1–39	5	0.1		Referent		39	0.6		Referent		2.00 (1.96–2.29)
40–59	56	1.0		2.60 (1.70–3.26)		284	3.8		1.76 (1.41–1.85)		3.80 (3.68–3.96)
>60	98	2.4		7.50 (5.00–9.42)		739	14.6		11.02 (8.86–11.51)		6.10 (6.26–6.67)
Other species											
Total	87	2.1	NA	NA		251	5.3	NA	NA		2.52 (2.16–2.89)
<1–39	11	0.1		Referent		50	0.7		Referent		7.00 (6.43–7.69)
40–59	35	0.5		3.24 (1.94–4.56)		79	1.2		2.21 (1.55–2.64)		2.40 (1.90–2.50)
>60	41	0.9		4.30 (2.58–5.99)		122	2.3		4.56 (3.26–5.34)		2.56 (2.35–2.83)
Unknown	139	NA				145	NA				

*Crude rate is cases per 100,000 population. ASR, age-standardized rate; IRR, incidence rate ratio; NA, not applicable. †Age standardized to the New Zealand population age structure at the 2013 census.

We recorded ethnicity for 2,508 (95.4%) notified cases during 2000‒2020 and compiled age-stratified and age-standardized rates of legionellosis for European, Māori, Pacific Peoples, and other ethnicities (Appendix Tables 4, 5). Focusing on age-standardized rates for the 2010*–*2020 period, the European ethnic group had the highest notification rate (3.9 cases/100,000 population), followed by Pacific Peoples (3.1 cases/100,000 population), Māori (2.8 cases/100,000 population), and other ethnic group persons (1.7 cases/100,000 population). The age-standardized rates increased for all ethnicities over the 2 time periods. A notable change in the second time period (2010*–*2020) was that the ethnic gradient toward higher rates in Europeans was reduced because rates had increased more markedly for Māori, Pacific Peoples, and other ethnic groups over that observation period (Table 1).

### Regional Distribution

**Figure 5:**
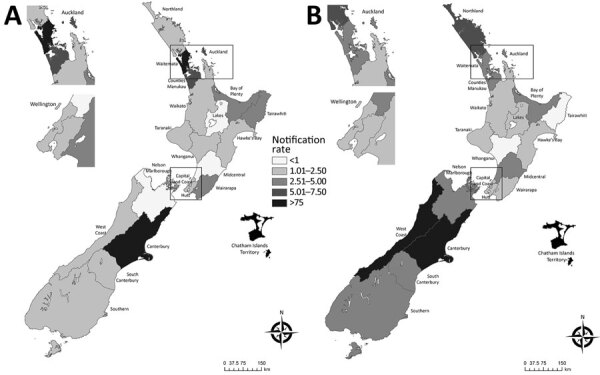
Geographic pattern of mean legionellosis notification rates (cases/100,000 population) by New Zealand District Health Board in study of increased incidence of legionellosis after improved diagnostic methods, New Zealand, 2000–2020. A) 2000–2009; B) 2010–2020. Insets show enlarged areas around the cities of Auckland and Wellington. Maps generated in ArcGIS version 10.8 (https://www.arcgis.com/index.html) by using District Health Board data (Appendix).

We compiled the rates of legionellosis incidence calculated for each district health board (DHB) area that had >5 diagnosed cases (divided into quintiles based on mean rate/100,000 population) for 2000–2009 and 2010–2020 (Figure 5; Appendix Table 6). The two highest quintiles were well above the mean national notifiable incidence rate of 2.7 cases/100,000 population. In the South Island, large changes in the legionellosis rate were observed on the West Coast (2.0 cases/100,000 population in 2000–2009 and 10.6 cases/100,000 population in 2010–2020), partly influenced by the small population size. The Canterbury DHBs of the South Island showed consistently high rates (9.1 cases/100,000 population in 2000–2009 and 9.8 cases/100,000 population in 2010–2020). In the North Island, large changes in the incidence rate were observed in Northland, with an observed increase in mean annual incidence from 2.0 cases/100,000 population in 2000–2009 to 6.0 cases/100,000 population in 2010–2020. Conversely, a decrease in incidence rates was observed across the 3 Auckland DHBs; the Central Auckland DHB had the largest decrease in mean annual incidence, from 5.5 cases/100,000 population in 2000–2009 to 3.3 cases/100,000 population in 2010–2020. Conversely, a decrease in incidence rates was observed across the 3 Auckland DHBs; the Central Auckland DHB had the largest decrease in mean annual incidence, from 5.5 cases/100,000 population in 2000–2009 to 3.3 cases/100,000 population in 2010–2020.

### Case Outcome

The hospitalization status was recorded on EpiSurv (https://surv.esr.cri.nz) for 95.8% (2,518/2,628) notified cases during 2000–2020. Of those case-patients, most (82.0%, 2,066/2,518) were hospitalized; 90 (3.6%) recorded unknown hospitalization status. The risk for hospitalization decreased over time: 91.4% (588/643 case-patients) were hospitalized during 2000–2009 but only 74.5% (14,78/1,985 case-patients) during 2010–2020. The highest percentage of hospitalized case-patients was >60 years of age for both periods, 82.3% (375 cases) in 2000–2009 and 81.1% (1,308 cases) in 2010–2020. The rate of hospitalization for legionellosis increased from 14.2 cases/100,000 population in 2000–2009 to 31.4 cases/100,000 population in 2010–2020.

A total of 61 deaths attributed to legionellosis were reported during 2000‒2020, giving an overall case-fatality risk (CFR) of 2.7% (range 0.4%‒8.9%). The CFR decreased from 4.0% (26 deaths/643 notified cases) during 2000–2009 to 1.8% (35 deaths/1985 notified cases) in 2010–2020. Throughout the study period, an increased CFR was consistently associated with advanced age and male sex. The increase in cases was associated with a marked decrease in CFR for *L. longbeachae* but little change for *L. pneumophila* (Appendix Table 7). The increase in case detection during 2010–2020 identified a larger number of less severe cases, which effectively increased the denominator of nonfatal cases and decreased the observed CFR by ≈60%. This effect was particularly marked for *L. longbeachae*, for which the CFR has decreased by 80% and is now markedly less than that observed for *L. pneumophila* (Appendix Table 7). The legionellosis mortality rate increased slightly between study periods, from 0.6 deaths/100,000 population in 2000–2009 to 0.7 deaths/100,000 population in 2010–2020.

### Risk Factors

The surveillance system routinely collects data on a range of environmental exposures reported by cases along with key host factors that are known to predispose to legionellosis (Table 2). An environmental exposure risk was reported for 1,744 (68.4%) of laboratory-identified cases recorded in the EpiSurv database during 2000‒2020. More detailed exposure data were available for the second decade and showed that 1,054 (41.4%) case-patients reported contact with compost/potting mix or soil during their incubation period (Table 2). A total of 155 (6.2%) of the number of reported notified case-patients had a history of overseas travel during the incubation period. Smoking and an immunosuppressive or debilitating condition were commonly reported by notified case-patients (Table 2).

**Table 2:** Increased incidence of legionellosis after improved diagnostic methods, showing risk factors associated with notified case-patients who had legionellosis and percentages reporting exposure, New Zealand, 2000–2020*

Risk factor	No. (%) cases		Odds ratio (95% CI), 2010–2020 compared with 2000–2009
	2000–2009	2010–2020	
Hospital-acquired	2 (0.3)	6 (0.2)	0.8 (0.2–3.8)
Overseas travel during incubation period	43 (6.7)	112 (4.4)	0.65 (0.45–0.93)
Contact with definite or suspected environmental source	307 (47.5)	1,437 (56.4)	1.43 (1.09–1.3)
Compost source contact	ID	1,054 (41.4)	ID
Water source contact	ID	91 (3.6)	ID
Smoker or ex-smoker	120 (18.6)	326 (12.8)	0.64 (0.51–0.81)
Preexisting immunocompromised or debilitating condition	174 (26.9)	666 (26.1)	0.97 (0.79–1.27)
Total	646 (100.0)	2,547 (100.0)	NA

*Percentages refer to notified case-patients who answered yes for the total number of notified cases for which a response was recorded. Some notified case-patients had >1 risk factor recorded. ID, incomplete data (no comparison could be made); NA, not applicable.

## Discussion

This study provides a comprehensive analysis of the epidemiology of legionellosis over the 21-year period of 2000–2020 in New Zealand by using notifications and national laboratory-based surveillance of *Legionella* cases. The study period saw a large increase in disease incidence driven by several factors that we investigated.

A major finding is the marked increase in the reported incidence of legionellosis from 2010 onward (Figure 1; Appendix Table 1). This increase is associated with improved case ascertainment, likely driven by increased clinical awareness of legionellosis and increased availability of specific laboratory testing for legionellosis. The marked increase in legionellosis notifications during 2015 and 2016 was caused by the LegiNZ prospective study, which provided a 12-month period (May 2015‒May 2016) of intensified surveillance (*11*). During that study, all lower respiratory samples from hospitalized notified-case patients who had suspected pneumonia were tested for *Legionella* spp. by PCR. An increase in case detection in 17 regions (Figure 5) was expected, with the national 86% increase more likely caused by historical underdiagnosis of the disease, rather than an increase in disease burden (*12*). For that reason, the legionellosis rate of 3.9 cases/100,000 during 2010–2020 probably provides a more valid estimate of the true population rate than seen previously; the higher rate of 5.4 cases/100,000 population detected by the LegiNZ study is likely to be particularly robust. Those rates put New Zealand above an estimated global mean rate of 2.8 cases/100,000 population (95% CI 2.7–2.9 cases/100,000 population) derived from the reported contribution of *Legionella* species to community-acquired pneumonia in multiple countries (*1*). The relatively small increase in the mortality rate of legionellosis during this period (from 0.6 deaths/100,000 population in 2000–2009 to 0.7 deaths/100,000 population in 2010–2020) is also consistent with the conclusion of greater case ascertainment of less severe cases being the main driver of the apparent increase in disease incidence during this period.

Unlike jurisdictions outside New Zealand that observed a temporary decrease in legionellosis at the beginning of the COVID-19 pandemic (*13*), rates for New Zealand did not appear to have been affected in 2020 (Figure 1). This finding suggests that environmental exposures to *Legionella* species may not have changed in New Zealand during this period, which could reflect the COVID-19 elimination strategy that enabled ordinary life to continue for most of that year, with only a few weeks under lockdown (*14*). The pandemic and its response had complex effects on the epidemiology of many infectious diseases and their surveillance. For example, studies undertaken in other jurisdictions outside New Zealand have identified greatly increased *Legionella* microbial contamination in building water systems (cooling towers) linked to extreme water stagnation caused by prolonged closures of commercial buildings, reinforcing the need for monitoring water and air conditioning systems (*15*).

The findings of this study have shown the dramatic shift in legionellosis diagnosis during a period when traditional techniques were largely replaced by molecular methods and UAT. However, a key limitation of UAT is that it cannot detect organisms other than *L. pneumophila* serogroup 1 (*16*); some authors have suggested that a total dependence on this diagnostic assay may miss up to 40% of legionellosis cases (*17*). Another limitation of UAT is that it does not generate material that can be used for typing methods. In New Zealand, the *Legionella* UAT has been used by several laboratories since 1998 but has decreased utility because of the high proportion of legionellosis caused by non–*L. pneumophila* species, such as *L. longbeachae* (Figure 3; Appendix Table 3). In this setting, a negative UAT result does not exclude legionellosis and necessitates further testing to elucidate either exclusion or inclusion. Because only 20.3% of the 2,675 cases diagnosed during the study period were caused by *L. pneumophila* serogroup 1 infection (Appendix Table 2), potentially 80% of cases could be missed if only UAT were used. A recent evaluation of the UAT for the diagnosis of *L. longbeachae* infection indicated a sensitivity of 59.1% and specificity of 82.2% (*18*). Further development of the assay should improve sensitivity to strengthen its application as a useful diagnosis tool, particularly in laboratories in which there is limited molecular testing capacity and because of the ease of specimen collection and rapidity of diagnosis (*18*).

During the 1990s, the drive for better diagnostic methods led to development of several PCRs. However, a combination of factors, including test cost, reagent quality issues, contamination problems, and the lack of trained and skilled staff, resulted in the initial slow adoption of molecular diagnostics for legionellosis (*19*). The technology has now matured, and since 2010, when some laboratories in New Zealand began routine molecular diagnostic testing for legionellosis, it has now become the method of first choice. This change was caused by the availability of more robust and sensitive assays for the detection of many difficult to diagnose diseases, in addition to legionellosis, and the overall reduction in test costs. Molecular testing for legionellosis has also been driven by its superior diagnostic utility compared with traditional methods because it enables detection of all *Legionella* species and can obtain a result within hours of sample collection (*20*).

The shift in laboratory methods during 2000–2020 has influenced the ability of routine surveillance to detect the contribution of species and serogroups. We observed a marked increase in *L. longbeachae* cases compared with other species, such as *L. pneumophila*, since 2010 (Figure 2; Appendix Table 2). We also observed a decrease in the identification of the serogroup for *Legionella* species caused by reductions in the use of the traditional methods, namely culture and serology, which risks gaps in surveillance information and can hinder cluster analysis and source tracing (Appendix Table 2) (*21*). This trend was caused by increased use of PCR testing alone that identifies the species, but not the serogroup of the *Legionella* species. No single laboratory test combines both optimal diagnostic accuracy with the ability to epidemiologically type the causative agent. To achieve this feature, a combination of molecular testing supported by culture, serologic testing, or both is required (*19*). In recent years, whole-genome sequencing has emerged as a major tool to support epidemiologic investigation (suspected clusters and outbreaks) of Legionnaires’ disease and for characterization of new strains, but this method still requires the culture isolation of the bacterium.

The results of this study show that the rates of legionellosis were highest in adults >60 years of age and in male notified case-patients, consistent with previously reported research (*6*). As the population ages in New Zealand, the burden of legionellosis is likely to continue to increase in the absence of effective measures to prevent or adequately control the risk for infection. Legionellosis rates were higher in persons from Europe compared with Māori, Pacific Peoples, and persons of other ethnicities during 2000–2009, even after age standardization. Those differences largely disappeared during 2010–2020, corresponding with increased case ascertainment. This pattern is different from that seen for other serious infectious diseases, for which rates are markedly higher for Māori and Pacific Peoples (*22*). Those unexpected differences need further investigation to see if there is systematic underdiagnosis of legionellosis across ethnic groups or if differences in exposure might explain the pattern seen.

This analysis found that infections caused by *L. longbeachae* increasingly dominated over those caused by *L. pneumophila* (Figure 2; Appendix Table 2). The largest contribution to this increase in *L. longbeachae* cases came from persons >60 years of age (Table 1). Early spring‒summer clusters of *L. longbeachae* infections are seen each year and might be linked to increased gardening activity in warmer months, which has been shown to provide several psychological, physical, and social benefits for older persons (*23*). In contrast, *L. pneumophila* infections appear to be spread evenly throughout the year, and transmission by aerosols containing contaminated water from cooling towers was the most identified source from outbreak investigations in New Zealand. Decreased incidence rates observed across the 3 Auckland DHBs between decades might reflect introduction of a bylaw in 2015 requiring owners to register their industrial wet cooling tower systems annually and monitor *Legionella* bacteria levels (*24*). In contrast, higher incidence rates in the Northland region might reflect the readiness of clinicians to consider testing for *Legionella* species in response to the national surveillance study (*11*).

Our study also provides data on major risk factors, exposures, and potential transmission settings. Few cases were classified as hospital acquired. A small percentage (6.2%) were classified as travel-associated, based on having a history of overseas travel during the incubation period. That percentage is lower than that for Europe, where 14.7% of detected Legionnaires’ disease cases in 2019 were linked to travel abroad, of which 79% were linked to overnight stays in hotels (*25*). This difference is observed despite New Zealand having among the highest per capita international travel rates in the world, with >3 million residents departing New Zealand in 2019 (*26*).

Our study has several limitations associated with the use of routinely collected surveillance data. The most critical limitation is the long-term underascertainment of legionellosis. This limitation has been partially corrected by using more sensitive molecular testing, resulting in a marked increase in measured rates of legionellosis during the 2010‒2020 period. A further limitation is the incomplete reporting of some variables.

More research on the epidemiology of legionellosis in New Zealand is warranted. The high percentage of hospitalizations (82.0%) reported during the study period means that those data can be analyzed to provide a useful basis to identify emerging issues and determine priorities for prevention. For example, the discharge data could be used to estimate the economic cost of hospitalized cases of legionellosis in New Zealand. It would also be useful to investigate the contribution of *Legionella* infection to the burden of community-acquired pneumonia of mild-to-moderate severity, which will often be treated empirically outside the hospital setting without any etiologic diagnosis. Proposed interventions to reduce the effect of legionellosis in New Zealand should also be evaluated after they are implemented to determine their efficacy.

Appendix Additional information on increased incidence of legionellosis after improved diagnostic methods, New Zealand, 2000–2020.
