# Supplementary material for: Increased Incidence of Legionellosis after Improved Diagnostic Methods, New Zealand, 2000–2020
Source: Emerg Infect Dis. 2023 Jun;29(6):1173–82. doi: 10.3201/eid2906.221598 (PMC10202871; doi:10.3201/eid2906.221598)
Supplement: Appendix — Additional information on increased incidence of legionellosis after improved diagnostic methods, New Zealand, 2000–2020. [file 22-1598-Techapp-s1.pdf]

Article DOI: https://doi.org/10.3201/eid2906.221598

*EID cannot ensure accessibility for supplementary materials supplied by authors. Readers who have difficulty accessing supplementary content should contact the authors for assistance.*

# Increased Incidence of Legionellosis after Improved Diagnostic Methods, New Zealand, 2000–2020

## Appendix

## Methods

### Surveillance Data

In New Zealand, notification is mandated under the *Health Act 1956*, which requires health professionals and all medical diagnostic laboratories (notification also became a legal requirement for all laboratories in December 2007 under the *Health Amendment Act 2006*) to inform their local medical officer of health of any notifiable disease that they suspect or diagnose. These notifications provide the basis for disease surveillance and public health action, and hence aid in the control of legionellosis and other infectious diseases in New Zealand. Notification data are captured at each Public Health Unit of District Health Boards via a secure web-based portal into a centralized database (EpiSurv), New Zealand's national database for notifiable disease surveillance.

Laboratory-based surveillance and laboratory testing are conducted on clinical specimens referred to the national Legionella Reference Laboratory (LRL) at the Institute of Environmental Science and Research (ESR). These clinical samples are submitted to LRL for confirmatory testing, identification, and typing with results aggregated for analysis as laboratory-identified cases.

The surveillance data are collated by ESR on behalf of the New Zealand Ministry of Health. Data extracted from EpiSurv include case demographics, clinical features, laboratory

identification of the causative agent, and hospitalization status. The case-fatality risk was calculated where legionellosis was reported as the underlying cause of death (on the death certificate), as a measure of disease severity.

**Population Data**

New Zealand's population of just over 5 million is ethnically diverse. In the 2018 census data, the European ethnic group accounted for 70.2%, Māori 16.1%, Asian 15.1%, Pacific Peoples 8.1%, and Middle Eastern/Latin American/African (MELAA) 1.5% of the total population (*1*). Consistent with most high- and middle-income countries, the proportion of elderly persons (≥65 years) in New Zealand is also increasing.

The denominator populations were obtained from the 2001, 2006, 2013 and 2018 New Zealand Censuses, using linear interpolation to provide estimates in the inter-census years; these rates were directly age-standardized to 2013 New Zealand census usually residential population (at 0–4, 5–9, 10–14, 15–19, 20–29, 30–39, 10-years age band, up to 70+ years old). To account for the fact that a person can indicate that they belong to more than one ethnic group on the legionellosis case report form and in the census form, a 'prioritized' classification of ethnicity in accordance with the Ministry of Health's protocols for both numerator and denominator was used. For the 2006 census, the 'Other' category was split into two groups: 'Middle Eastern/Latin American/African' (MELAA); and 'Other Ethnicity'. Four prioritized ethnic groups were used in this analysis. They included the Māori ethnic group at the top of the hierarchy, followed by Pacific peoples (Cook Island, Samoan, Tongan, Niuean, Tokelauan, Tuvaluan, Fijian) 'Other Ethnicity' (Latin American, Asian, Middle Eastern, African) and European (including New Zealander and other European combined).

Ethical approval was not required for this stud because persons were not identifiable in the data, and only aggregated data were analyzed and presented.

**Geographic Data**

Maps were generated to display rates by District Health Board (DHB) area over two time periods (2000–2009 and 2010–2020), using ArcGIS software (version 10.8; ESRI, USA). In New Zealand, all 20 DHBs are responsible for providing of health and disability services within a defined geographic area. The Boards have a mean population of 219 000 and range from 32 000 (largely rural) to 629 000 (metropolitan).

**Reference**

1. Ministry of Health. Statistics New Zealand. Estimates and projections, 2020; 2017 [cited 2023 Apr 12]. https://www.stats.govt.nz

**Appendix Table 1.** Legionellosis notifications and laboratory-identified cases by year, 2000–2020 with year-on-year % change

| Year | 2000 | 2001 | 2002 | 2003 | 2004 | 2005 | 2006 | 2007 | 2008 | 2009 | 2010 | 2011 | 2012 | 2013 | 2014 | 2015 | 2016 | 2017 | 2018 | 2019 | 2020 |
|---|---|---|---|---|---|---|---|---|---|---|---|---|---|---|---|---|---|---|---|---|---|
| **Notified cases** | 61 | 46 | 49 | 77 | 62 | 85 | 52 | 64 | 73 | 74 | 173 | 158 | 149 | 155 | 125 | 254 | 247 | 221 | 174 | 169 | 160 |
| **Change from previous year (%)** | 29.5 | −32.6 | 6.1 | **36.4** | −24.2 | 27.1 | **-63.5** | 18.8 | 12.3 | 1.4 | **57.2** | −9.5 | −6.0 | 3.9 | −24.0 | **50.8** | −2.8 | −11.8 | −27.0 | −3.0 | −5.6 |
| **Laboratory identified cases** | 56 | 56 | 48 | 81 | 75 | 83 | 54 | 72 | 73 | 77 | 178 | 160 | 152 | 151 | 135 | 251 | 248 | 221 | 182 | 161 | 161 |
| **Change from previous year (%)** | 16.1 | 0.0 | −16.7 | **40.7** | −5.3 | 9.6 | **-53.7** | 25.0 | 1.4 | 5.2 | **56.7** | −11.3 | −5.3 | −0.7 | −11.9 | **46.2** | −1.2 | −11.8 | −17.6 | −11.5 | 0.0 |

**Appendix Table 2.** *Legionella* strains for laboratory-identified clinical cases, 2000–2020 (N = 2675)

| *Legionella* species and serogroup (%) | 2000 | 2001 | 2002 | 2003 | 2004 | 2005 | 2006 | 2007 | 2008 | 2009 | 2010 | 2011 | 2012 | 2013 | 2014 | 2015 | 2016 | 2017 | 2018 | 2019 | 2020 | Percentage (%) |
|---|---|---|---|---|---|---|---|---|---|---|---|---|---|---|---|---|---|---|---|---|---|---|
| ***L. longbeachae*** | **28 (50)** | **31 (55.4)** | **12 (25.0)** | **26 (32.1)** | **23 (30.7)** | **25 (30.1)** | **19 (35.2)** | **26 (36.1)** | **38 (52.1)** | **32 (41.6)** | **72 (40.4)** | **70 (43.8)** | **78 (51.3)** | **76 (50.3)** | **73 (54.1)** | **131 (52.2)** | **167 (67.3)** | **152 (68.8)** | **91 (50.0)** | **98 (60.9)** | **106 (65.8)** | **51.0** |
| ***L. longbeachae* sg 1** | 22 (78.6) | 26 (83.9) | 8 (66.7) | 8 (30.8) | 8 (34.8) | 14 (56.0) | 6 (31.6) | 8 (30.8) | 19 (50.0) | 23 (71.9) | 34 (47.2) | 29 (41.4) | 20 (25.6) | 20 (26.3) | 19 (26.0) | 60 (45.8) | 70 (41.9) | 67 (44.1) | 42 (46.2) | 46 (46.9) | 43 (40.6) | 22.1 |
| ***L. longbeachae* sg 2** | 2 (7.1) | 0 | 1 (8.3) | 3 (11.5) | 3 (13.0) | 4 (16.0) | 1 (5.3) | 7 (26.9) | 1 (2.6) | 4 (12.5) | 5 (6.9) | 3 (4.3) | 13 (16.7) | 9 (11.8) | 10 (13.7) | 15 (11.5) | 11 (6.6) | 3 (1.9) | 2 (2.2) | 5 (5.1) | 0 | 3.8 |
| ***L. longbeachae* sg 1 and 2 *** | 0 | 0 | 0 | 0 | 0 | 0 | 0 | 0 | 0 | 0 | 0 | 0 | 0 | 0 | 0 | 0 | 1 (0.6) | 0 | 0 | 0 | 0 | 0.0 |
| ***L. longbeachae/L. bozemanae* sg 1** | 0 | 0 | 0 | 1 (3.8) | 0 | 1 (4.0) | 0 | 0 | 0 | 0 | 0 | 0 | 0 | 0 | 0 | 0 | 0 | 0 | 1 (1.1) | 0 | 2 (1.9) | 0.3 |
| ***L. longbeachae/L. jordanis*** | 0 | 0 | 0 | 0 | 0 | 0 | 0 | 0 | 0 | 0 | 1 (1.4) | 0 | 0 | 0 | 0 | 0 | 0 | 2 (1.3) | 0 | 0 | 0 | 0.04 |
| ***L. longbeachae/L. dumoffii*** | 0 | 0 | 0 | 0 | 0 | 0 | 0 | 0 | 0 | 0 | 0 | 0 | 0 | 0 | 0 | 0 | 0 | 2 (1.3) | 1 (1.1) | 0 | 0 | 0.11 |
| ***L. longbeachae* sg not determined** | 4 (14.3) | 5 (16.1) | 3 (25.0) | 14 (53.8) | 12 (52.2) | 6 (24.0) | 3 (15.8) | 11 (42.3) | 18 (47.4) | 5 (15.6) | 32 (44.4) | 38 (54.3) | 45 (57.7) | 47 (61.8) | 44 (60.3) | 56 (42.7) | 85 (50.9) | 78 (51.3) | 45 (49.5) | 47 (48.0) | 61 (57.5) | 24.7 |
| ***L. pneumophila*** | **15 (26.8)** | **9 (16.1)** | **18 (37.5)** | **29 (35.8)** | **30 (40.0)** | **49 (59.0)** | **31 (57.4)** | **29 (40.3)** | **24 (32.9)** | **34 (44.2)** | **51 (28.7)** | **48 (30.0)** | **51 (33.6)** | **42 (27.8)** | **46 (34.1)** | **73 (29.1)** | **51 (20.6)** | **53 (23.9)** | **61 (33.5)** | **50 (31.1)** | **44 (27.3)** | **31.2** |
| ***L. pneumophila* sg 1** | 5 (33.3) | 2 (22.2) | 9 (50.0) | 5 (17.2) | 19 (63.3) | 40 (81.6) | 22 (70.9) | 17 (58.6) | 20 (83.3) | 25 (73.5) | 32 (62.7) | 38 (79.2) | 39 (76.5) | 31 (73.8) | 23 (50.0) | 44 (60.3) | 29 (56.9) | 34 (64.2) | 49 (80.3) | 31 (62.0) | 29 (65.9) | 20.3 |
| ***L. pneumophila* sg 2** | 0 | 1 (11.1) | 0 | 8 (27.6) | 1 (3.3) | 1 (2.0) | 0 | 1 (3.4) | 1 (4.2) | 2 (5.9) | 2 (3.9) | 3 (6.3) | 1 (1.9) | 0 | 1 (2.2) | 1 (1.4) | 3 (5.9) | 1 (1.9) | 2 (3.3) | 2 (4.0) | 0 | 1.2 |
| ***L. pneumophila* sg 3** | 0 | 0 | 1 (5.6) | 0 | 0 | 0 | 0 | 0 | 0 | 0 | 0 | 0 | 1 (1.9) | 1 (2.4) | 1 (2.2) | 1 (1.4) | 0 | 0 | 0 | 0 | 0 | 0.2 |
| ***L. pneumophila* sg 4** | 1 (6.7) | 0 | 0 | 2 (6.9) | 3 (10.0) | 2 (4.1) | 4 (12.9) | 0 | 0 | 3 (8.8) | 8 (15.7) | 2 (4.2) | 2 (3.9) | 1 (2.4) | 2 (4.3) | 1 (1.4) | 1 (1.9) | 1 (1.9) | 1 (1.6) | 1 (2.0) | 0 | 1.3 |
| ***L. pneumophila* sg 5** | 3 (20.0) | 0 | 0 | 0 | 0 | 0 | 1 (3.2) | 1 (3.4) | 1 (4.2) | 0 | 0 | 0 | 0 | 1 (2.4) | 0 | 3 (4.1) | 0 | 1 (1.9) | 0 | 0 | 1 (2.3) | 0.5 |
| ***L. pneumophila* sg 6** | 0 | 0 | 0 | 0 | 0 | 0 | 0 | 0 | 1 (4.2) | 1 (2.9) | 4 (7.8) | 0 | 0 | 0 | 1 (2.2) | 0 | 1 (1.9) | 0 | 0 | 0 | 1 (2.3) | 0.3 |
| ***L. pneumophila* sg 7** | 0 | 0 | 0 | 0 | 0 | 0 | 0 | 0 | 0 | 0 | 0 | 0 | 0 | 0 | 0 | 0 | 0 | 1 (1.9) | 1 (1.6) | 1 (2.0) | 0 | 0.1 |
| ***L. pneumophila* sg 8** | 0 | 0 | 1 (5.6) | 0 | 0 | 0 | 0 | 1 (3.4) | 0 | 0 | 0 | 0 | 0 | 0 | 0 | 2 (2.7) | 0 | 1 (1.9) | 0 | 0 | 0 | 0.2 |
| ***L. pneumophila* sg 10** | 0 | 0 | 0 | 1 (3.4) | 0 | 0 | 0 | 0 | 0 | 0 | 1 (1.9) | 0 | 0 | 2 (4.8) | 0 | 0 | 0 | 1 (1.9) | 0 | 0 | 0 | 0.2 |
| ***L. pneumophila* sg 11** | 0 | 1 (11.1) | 0 | 0 | 0 | 0 | 0 | 0 | 0 | 0 | 0 | 0 | 0 | 0 | 1 (2.2) | 0 | 0 | 0 | 0 | 0 | 0 | 0.1 |
| ***L. pneumophila* sg 12** | 0 | 1 (11.1) | 3 (16.7) | 8 (27.6) | 1 (3.3) | 0 | 0 | 3 (10.3) | 1 (4.2) | 0 | 4 (7.8) | 3 (6.3) | 4 (7.8) | 3 (7.1) | 12 (26.1) | 13 (17.8) | 8 (15.7) | 3 (5.7) | 1 (1.6) | 0 | 0 | 2.5 |
| ***L. pneumophila* sg 13** | 0 | 2 (22.2) | 1 (5.6) | 0 | 0 | 0 | 0 | 1 (3.4) | 0 | 1 (2.9) | 0 | 2 (4.2) | 0 | 1 (2.4) | 1 (2.2) | 0 | 1 (1.9) | 1 (1.9) | 1 (1.6) | 0 | 1 (2.3) | 0.5 |
| ***L. pneumophila* sg 14** | 0 | 0 | 0 | 3 (10.3) | 0 | 0 | 0 | 0 | 0 | 0 | 0 | 0 | 0 | 0 | 0 | 0 | 0 | 0 | 0 | 0 | 1 (2.3) | 0.1 |
| ***L. pneumophila* sg 15** | 0 | 0 | 0 | 0 | 1 (3.3) | 0 | 0 | 2 (6.9) | 0 | 0 | 0 | 0 | 0 | 0 | 1 (2.2) | 1 (1.4) | 0 | 0 | 0 | 0 | 0 | 0.2 |
| ***L. pneumophila* strain 97–2898** | 0 | 0 | 0 | 0 | 0 | 0 | 0 | 0 | 0 | 0 | 0 | 0 | 0 | 0 | 0 | 0 | 0 | 0 | 1 (1.6) | 0 | 0 | 0.0 |
| ***L. pneumophila* strain 91–033** | 0 | 0 | 0 | 0 | 0 | 0 | 0 | 0 | 0 | 0 | 0 | 0 | 0 | 0 | 0 | 0 | 0 | 1 (1.9) | 0 | 0 | 0 | 0.0 |
| ***L. pneumophila* sg not determined** | 6 (40.0) | 2 (22.2) | 2 (11.1) | 2 (6.9) | 5 (16.7) | 6 (12.2) | 4 (12.9) | 3 (10.3) | 0 | 0 | 0 | 0 | 4 (7.8) | 2 (4.8) | 3 (6.5) | 7 (9.6) | 8 (15.7) | 8 (15.1) | 6 (9.8) | 15 (30.0) | 11 (25.0) | 3.5 |
| **Other *Legionella* species with id (%)** | **9 (16.1)** | **11 (19.6)** | **18 (37.5)** | **23 (28.4)** | **18 (24.0)** | **7 (8.4)** | **6 (11.1)** | **14 (19.4)** | **11 (15.1)** | **11 (14.3)** | **37 (20.8)** | **30 (18.8)** | **20 (13.2)** | **22 (14.6)** | **14 (10.4)** | **33 (13.1)** | **21 (8.5)** | **13 (5.9)** | **19 (10.4)** | **12 (7.5)** | **7 (4.3)** | **13.5** |
| ***L. anisa*** | 1 (11.1) | 0 | 0 | 1 (4.3) | 0 | 0 | 1 (16.7) | 0 | 0 | 0 | 0 | 0 | 0 | 0 | 0 | 2 (6.1) | 0 | 0 | 0 | 0 | 0 | 0.2 |
| ***L. anisa/L. bozemanae* sg 1** | 0 | 0 | 0 | 0 | 0 | 0 | 0 | 0 | 0 | 0 | 0 | 0 | 0 | 0 | 0 | 0 | 0 | 0 | 0 | 0 | 0 | 0.0 |
| ***L. bozemanae* sg 1** | 1 (11.1) | 0 | 3 (16.7) | 3 (13.0) | 3 (16.7) | 2 (28.6) | 0 | 1 (7.1) | 2 (18.2) | 1 (9.1) | 7 (18.9) | 1 (3.3) | 0 | 4 (18.2) | 0 | 4 (12.1) | 3 (14.3) | 1 (7.7) | 2 (10.5) | 2 (16.7) | 1 (14.3) | 1.5 |
| ***L. bozemanae* sg 2** | 3 (33.3) | 0 | 0 | 1 (4.3) | 1 (5.6) | 0 | 0 | 0 | 0 | 0 | 1 (2.7) | 2 (6.7) | 0 | 0 | 0 | 0 | 1 (4.8) | 1 (7.7) | 0 | 0 | 0 | 0.4 |
| ***L. bozemanae* sg not determined** | 0 | 0 | 2 (11.1) | 1 (4.3) | 0 | 0 | 0 | 0 | 0 | 0 | 0 | 1 (3.3) | 0 | 0 | 0 | 0 | 0 | 0 | 0 | 0 | 0 | 0.1 |
| ***L. dumoffii*** | 0 | 6 (54.5) | 3 (16.7) | 2 (8.7) | 3 (16.7) | 1 (14.3) | 3 (50.0) | 1 (7.1) | 5 (45.4) | 5 (45.4) | 13 (35.1) | 12 (40.0) | 5 (25.0) | 3 (13.6) | 2 (14.3) | 5 (15.2) | 2 (9.5) | 3 (23.1) | 3 (15.8) | 4 (33.3) | 0 | 3.0 |
| ***L. feeleii* sg 1** | 0 | 0 | 1 (5.6) | 0 | 1 (5.6) | 0 | 0 | 0 | 1 (9.1) | 0 | 2 (5.4) | 0 | 0 | 0 | 0 | 1 (3.0) | 0 | 0 | 1 (5.3) | 0 | 0 | 0.3 |
| ***L. gormaii*** | 2 (22.2) | 3 (27.3) | 4 (22.2) | 0 | 4 (22.2) | 3 (42.9) | 1 (16.7) | 3 (21.4) | 2 (18.2) | 2 (18.2) | 2 (5.4) | 2 (6.7) | 0 | 2 (9.1) | 1 (7.1) | 3 (9.1) | 0 | 1 (7.7) | 0 | 0 | 0 | 1.3 |
| ***L. hackeliae*** | 0 | 0 | 3 (16.7) | 7 (30.4) | 1 (5.6) | 1 (14.3) | 0 | 0 | 0 | 0 | 0 | 0 | 0 | 0 | 0 | 0 | 0 | 0 | 0 | 0 | 0 | 0.6 |
| ***L. harrisonii* sp. nov.** | 0 | 0 | 0 | 0 | 0 | 0 | 0 | 0 | 0 | 0 | 0 | 0 | 0 | 1 (4.5) | 0 | 0 | 0 | 0 | 2 (10.5) | 1 (8.3) | 0 | 0.2 |
| ***L. jordanis*** | 0 | 0 | 1 (5.6) | 1 (4.3) | 1 (5.6) | 0 | 0 | 2 (14.3) | 0 | 0 | 4 (10.8) | 3 (10.0) | 4 (20.0) | 1 (4.5) | 1 (7.1) | 4 (12.1) | 1 (4.8) | 0 | 2 (10.5) | 0 | 1 (14.3) | 1.0 |
| ***L. maceachernii*** | 0 | 0 | 0 | 0 | 0 | 0 | 0 | 0 | 0 | 0 | 0 | 0 | 0 | 0 | 0 | 0 | 0 | 0 | 0 | 1 (8.3) | 0 | 0.0 |
| ***L. micdadei*** | 2 (22.2) | 2 (18.2) | 1 (5.6) | 7 (30.4) | 4 (22.2) | 0 | 0 | 5 (35.7) | 1 (9.1) | 2 (18.2) | 6 (16.2) | 3 (10.0) | 9 (45.0) | 9 (40.9) | 8 (57.1) | 9 (27.3) | 5 (23.8) | 1 (7.7) | 5 (26.3) | 1 (8.3) | 2 (28.6) | 3.2 |
| ***L. oakridgensis*** | 0 | 0 | 0 | 0 | 0 | 0 | 0 | 0 | 0 | 0 | 0 | 0 | 0 | 0 | 0 | 0 | 2 (2.5) | 0 | 0 | 0 | 0 | 0.1 |

| *Legionella* species and serogroup (%) | 2000 | 2001 | 2002 | 2003 | 2004 | 2005 | 2006 | 2007 | 2008 | 2009 | 2010 | 2011 | 2012 | 2013 | 2014 | 2015 | 2016 | 2017 | 2018 | 2019 | 2020 | Percentage (%) |
|---|---|---|---|---|---|---|---|---|---|---|---|---|---|---|---|---|---|---|---|---|---|---|
| ***L. sainthelensi*** | 0 | 0 | 0 | 0 | 0 | 0 | 1 (16.7) | 2 (14.3) | 0 | 1 (9.1) | 2 (5.4) | 6 (20.0) | 2 (10.0) | 2 (9.1) | 2 (14.3) | 5 (15.2) | 5 (23.8) | 5 (38.5) | 4 (21.1) | 2 (16.7) | 3 (42.9) | 1.5 |
| ***L. wadsworthii*** | 0 | 0 | 0 | 0 | 0 | 0 | 0 | 0 | 0 | 0 | 0 | 0 | 0 | 0 | 0 | 0 | 0 | 1 (7.7) | 0 | 0 | 0 | 0.0 |
| ***Legionella* strain D5382** | 0 | 0 | 0 | 0 | 0 | 0 | 0 | 0 | 0 | 0 | 0 | 0 | 0 | 0 | 0 | 0 | 2 (9.5) | 0 | 0 | 0 | 0 | 0.1 |
| ***Legionella* species unidentified** | **4 (7.1)** | **5 (8.9)** | **0** | **3 (3.7)** | **2 (2.7)** | **2 (2.4)** | **3 (5.6)** | **3 (4.2)** | **0** | **2 (2.6)** | **18 (10.1)** | **12 (7.5)** | **3 (1.9)** | **11 (7.3)** | **2 (1.5)** | **14 (5.6)** | **9 (3.6)** | **3 (1.4)** | **11 (6.0)** | **2 (1.2)** | **4 (2.5)** | **4.3** |
| **Total** | **56** | **56** | **48** | **81** | **75** | **83** | **54** | **72** | **73** | **77** | **178** | **160** | **152** | **151** | **135** | **251** | **248** | **221** | **182** | **161** | **161** | **100** |

⁎ = dual infection

**Appendix Table 3.** Number of laboratory-identified cases by initial method of diagnosis by year, 2000–2020*

| | **Culture only** | **LUAT** | | **Molecular** | | **Serology only** | | | | | | | |
|---|---|---|---|---|---|---|---|---|---|---|---|---|---|
| **Year** | **Culture only (%)** | **UAT only (%)** | **UAT plus other (%)** | **PCR only (%)** | **PCR plus other (%)** | **SC/4fr (%)** | **2fr/stable High Titer (%)** | **Single High Titer (%)** | **Annual Total** | **Annual Confirmed (%)** | **Annual crude incidence rate (confirmed)** | **Annual Probable (%)** | **Annual crude incidence rate (probable)** |
| 2000 | 8 (14.3) | 1 (1.8) | 0 | 0 | 7 (12.5) | 17 (30.4) | 15 (26.8) | 8 (14.3) | 56 | 33 (58.9) | 0.9 | 23 (41.1) | 0.6 |
| 2001 | 7 (12.5) | 1 (1.8) | 0 | 0 | 1 (1.8) | 18 (32.1) | 22 (39.3) | 7 (12.5) | 56 | 27 (48.2) | 0.7 | 29 (51.8) | 0.7 |
| 2002 | 4 (8.3) | 0 | 5 (10.4) | 0 | 1 (2.1) | 15 (31.3) | 18 (37.5) | 5 (10.4) | 48 | 25 (52.1) | 0.6 | 23 (47.9) | 0.6 |
| 2003 | 5 (6.2) | 1 (1.2) | 0 | 2 (2.5) | 5 (6.2) | 23 (28.4) | 33 (40.7) | 12 (14.8) | 81 | 36 (44.4) | 0.9 | 45 (55.6) | 1.1 |
| 2004 | 8 (10.7) | 1 (1.3) | 1 (1.3) | 2 (2.7) | 1 (1.3) | 20 (26.7) | 30 (40.0) | 12 (16.0) | 75 | 33 (44.0) | 0.8 | 42 (56.0) | 1.0 |
| 2005 | 8 (9.6) | 17 (20.5) | 7 (8.4) | 2 (2.4) | 1 (1.2 | 12 (14.5) | 25 (30.1) | 11 (13.6) | 83 | 47 (56.6) | 1.3 | 36 (43.4) | 0.9 |
| 2006 | 3 (5.6) | 1 (1.9) | 7 (13.0) | 2 (3.7) | 1 (1.9) | 11 (20.4) | 19 (35.2) | 10 (18.5) | 54 | 25 (46.3) | 0.6 | 29 (53.7) | 0.7 |
| 2007 | 7 (9.7) | 1 (1.4) | 4 (5.6) | 1 (1.4) | 3 (4.2) | 22 (30.6) | 29 (40.3) | 5 (6.9) | 72 | 38 (52.8) | 0.9 | 34 (47.2) | 0.8 |
| 2008 | 12 (16.4) | 5 (6.8) | 6 (8.2) | 2 (2.7) | 7 (9.6) | 15 (20.5) | 23 (31.5) | 3 (0.4) | 73 | 47 (64.4) | 1.1 | 26 (35.6) | 0.6 |
| 2009 | 8 (10.4) | 12 (15.6) | 9 (11.7) | 0 | 9 (11.7) | 16 (20.8) | 20 (26.0) | 3 (3.9) | 77 | 54 (70.1) | 1.3 | 23 (29.9) | 0.5 |
| 2010 | 4 (2.2) | 11 (6.2) | 11 (6.2) | 16 (9.0) | 22 (12.4) | 36 (20.2) | 57 (32.0) | 21 (11.8) | 178 | 100 (56.2) | 2.3 | 78 (43.8) | 1.8 |
| 2011 | 0 | 7 (4.4) | 16 (10.0) | 16 (10.0) | 33 (20.6) | 31 (19.4) | 50 (31.3) | 7 (4.4) | 160 | 103 (64.4) | 2.3 | 57 (35.6) | 1.3 |
| 2012 | 0 | 10 (6.6) | 23 (15.1) | 27 (17.8) | 22 (14.5) | 19 (12.5) | 35 (23.0) | 16 (10.5) | 152 | 101 (66.4) | 2.3 | 51 (33.5) | 1.2 |
| 2013 | 0 | 10 (6.6) | 17 (11.3) | 34 (22.5) | 29 (19.2) | 21 (13.9) | 35 (23.2) | 5 (3.3) | 151 | 111 (73.5) | 2.5 | 40 (26.5) | 0.9 |
| 2014 | 0 | 10 (7.4) | 11 (8.1) | 30 (22.2) | 27 (20.0) | 17 (12.6) | 30 (22.2) | 10 (7.4) | 135 | 95 (70.4) | 2.1 | 40 (29.6) | 0.9 |
| 2015 | 1 (0.4) | 9 (3.6) | 25 (10.0) | 59 (23.6) | 66 (26.4) | 33 (13.2) | 36 (14.4) | 21 (8.4) | 250 | 193 (77.2) | 4.2 | 57 (22.8) | 1.2 |
| 2016 | 0 | 0 | 15 (6.0) | 93 (37.5) | 66 (26.6) | 36 (14.5) | 33 (13.3) | 5 (2.0) | 248 | 210 (84.7) | 4.5 | 38 (15.3) | 0.8 |
| 2017 | 1 (0.5) | 13 (5.9) | 15 (6.8) | 84 (38.0) | 54 (24.4) | 25 (11.3) | 22 (10.0) | 7 (3.2) | 221 | 192 (86.9) | 4.0 | 29 (13.1) | 0.6 |
| 2018 | 2 (1.1) | 28 (15.4) | 19 (10.4) | 58 (31.9) | 31 (17.0) | 32 (17.6) | 11 (6.0) | 1 (0.5) | 182 | 170 (93.4) | 3.5 | 12 (6.6) | 0.2 |
| 2019 | 0 | 17 (10.5) | 7 (4.3) | 91 (56.5) | 23 (14.2) | 10 (6.2) | 12 (7.4) | 1 (0.6) | 161 | 148 (91.9) | 3.0 | 13 (8.0) | 0.3 |
| 2020 | 0 | 15 (9.3) | 9 (5.6) | 87 (54.0) | 31 (19.3) | 11 (6.8) | 6 (3.7) | 2 (1.2) | 161 | 153 (95.0) | 3.2 | 8 (5.0) | 0.2 |
| **Total** | **78 (2.9)** | **170 (6.4)** | **207 (7.7)** | **607 (22.7)** | **440 (16.4)** | **440 (16.4)** | **561 (21.0)** | **172 (7.4)** | **2675** | **1942 (72.6)** | | **733 (27.4)** | |

*† includes culture and or molecular and or serology; ‡: includes culture &/or UAT &/or serology. Note each laboratory identified case has only been counted once. SC = seroconversion; 4fr = 4-fold titer rise; 2fr = 2-fold titer rise. Probable cases include 561+172 = 733.

*Rate per 100 000 population
# Rate per 100 000 population, age-standardized to the New Zealand population age-structure at the 2013 census
IRR = incidence rate ratio, compared with European rate, age standardized to the New Zealand population age-structure at the 2013 census

**Appendix Table 4.** Legionellosis number and incidence rate (notified cases) by ethnicity and age group (prioritized), New Zealand, 2000–2009

| | Māori | | | Pacific Peoples | | | Other Ethnicity | | | European | | |
|---|---|---|---|---|---|---|---|---|---|---|---|---|
| | 2000–2009 | | | 2000–2009 | | | 2000–2009 | | | 2000–2009 | | |
| Aged band | No | Pop | Rate* | No | Pop | Rate* | No | Pop | Rate* | No | Pop | Rate* |
| 0–4 | 1 | 65 958 | 0.2 | 0 | 25 134 | 0 | 0 | 59 595 | 0 | 0 | 124 404 | 0 |
| /5–9 | 1 | 66 396 | 0.2 | 1 | 25 368 | 0.4 | 0 | 62 340 | 0 | 0 | 132 408 | 0 |
| 10–14 | 0 | 66 390 | 0 | 0 | 25 020 | 0 | 0 | 65 322 | 0 | 1 | 149 223 | 0.1 |
| 15–19 | 0 | 58 344 | 0 | 0 | 22 854 | 0 | 1 | 65 340 | 0.2 | 1 | 153 717 | 0.1 |
| 20–29 | 4 | 80 610 | 0.5 | 1 | 34 638 | 0.3 | 1 | 150 300 | 0.1 | 13 | 247 842 | 0.5 |
| 30–39 | 6 | 77 847 | 0.8 | 5 | 33 690 | 1.5 | 2 | 151 791 | 0.1 | 33 | 314 730 | 1.0 |
| 40–49// | 4 | 69 024 | 0.6 | 4 | 27 204 | 1.5 | 2 | 158 523 | 0.1 | 87 | 352 290 | 2.5 |
| /50–59 | 6 | 42 687 | 1.4 | 0 | 17 043 | 0 | 3 | 119 442 | 0.3 | 115 | 306 990 | 3.7 |
| 60–69 | 3 | 22 947 | 1.3 | 2 | 9 645 | 2.1 | 2 | 70 539 | 0.3 | 113 | 224 982 | 5.0 |
| 70+ | 2 | 12 888 | 1.6 | 0 | 5 700 | 0 | 6 | 49 335 | 1.2 | 154 | 279 081 | 5.5 |
| **Total** | **27** | **565 091** | **0.5** | **13** | **226 296** | **0.6** | **17** | **952 527** | **0.2** | **517** | **2 285 667** | **2.3** |
| Age standardized rate# | | | **0.7 (0.4–1.1)** | | | **0.7 (0.3–1.1)** | | | **0.2 (0.1–0.4)** | | | **2.1 (1.9–2.3)** |
| IRR (95% CI) | | | **0.35 (0.22–0.55)** | | | **0.32 (0.17–0.58)** | | | **0.11 (0.07–0.19)** | | | **1.0** |

*Rate per 100 000 population
# Rate per 100 000 population, age-standardized to the New Zealand population age-structure at the 2013 census
IRR = incidence rate ratio, compared with European

**Appendix Table 5.** Legionellosis number and incidence rate (notified cases) by ethnicity and age group (prioritized), New Zealand, 2010–2020

| | Māori | | | Pacific Peoples | | | Other Ethnicity | | | European | | |
|---|---|---|---|---|---|---|---|---|---|---|---|---|
| | 2010–2020 | | | 2010–2020 | | | 2010–2020 | | | 2010–2020 | | |
| Aged band | No | Pop | Rate* | No | Pop | Rate* | No | Pop | Rate* | No | Pop | Rate* |
| 0–4 | 2 | 81207 | 0.2 | 0 | 42255 | 0 | 0 | 60360 | 0 | 2 | 194130 | 0.1 |
| 5–9 | 1 | 87822 | 0.1 | 0 | 45531 | 0 | 0 | 55101 | 0 | 4 | 217206 | 0.2 |
| 10–14 | 1 | 79758 | 0.1 | 0 | 40371 | 0 | 0 | 45567 | 0 | 2 | 210216 | 0.1 |
| 15–19 | 0 | 71079 | 0 | 1 | 38247 | 0.2 | 2 | 48558 | 0.4 | 6 | 202020 | 0.3 |
| 20–29 | 4 | 122067 | 0.3 | 6 | 65505 | 0.8 | 5 | 157311 | 0.3 | 19 | 404349 | 0.4 |
| 30–39 | 9 | 90480 | 0.9 | 6 | 46323 | 1.2 | 7 | 164067 | 0.4 | 54 | 367321 | 1.3 |
| 40–49 | 22 | 88578 | 2.3 | 12 | 40659 | 2.7 | 11 | 99210 | 1.0 | 155 | 428979 | 3.3 |
| 50–59 | 42 | 78984 | 4.8 | 16 | 32298 | 4.5 | 25 | 71571 | 3.2 | 285 | 457368 | 5.7 |
| 60–69 | 36 | 47994 | 6.8 | 17 | 18858 | 8.2 | 23 | 48033 | 4.4 | 452 | 389742 | 10.5 |
| 70+ | 32 | 27873 | 10.4 | 14 | 12141 | 10.5 | 20 | 28176 | 6.5 | 641 | 426426 | 13.7 |
| **Total** | **149** | **775 836** | **1.7** | **72** | **381 642** | **1.7** | **93** | **777 954** | **1.1** | **1620** | **194130** | **4.5** |
| **Age standardized rate#** | | | **2.8 (1.4–3.3)** | | | **3.1 (2.3–3.9)** | | | **1.7 (1.4–2.1)** | | | **3.9 (3.7–4.1)** |
| **IRR (95% CI)** | | | **0.73 (0.61–0.88)** | | | **0.80 (0.62–1.03)** | | | **0.45 (0.36–0.56)** | | | **1.0** |

*Rate per 100 000 population
# Rate per 100 000 population, age-standardized to the New Zealand population age-structure at the 2013 census
IRR = incidence rate ratio, compared with European rate, age standardized to the New Zealand population age-structure at the 2013 census

**Appendix Table 6.** Legionellosis number (notified) and incidence rate by District Health Board, New Zealand, 2000–2020

| | **2000–2009** | | **2010–2020** | |
|---|---|---|---|---|
| | **Total** | | **Total** | |
| **District Health Board** | Number | Rate | Number | Rate |
| Auckland | 66 | 5.5 | 149 | 3.3 |
| Bay of Plenty | 47 | 4.1 | 122 | 4.8 |
| Canterbury | 103 | 9.1 | 568 | 9.8 |
| Capital and Coast | 44 | 2.2 | 42 | 1.5 |
| Counties Manukau | 58 | 5.1 | 205 | 3.5 |
| Hawke's Bay | 21 | 1.9 | 31 | 1.7 |
| Hutt Valley | 30 | 2.3 | 33 | 2.1 |
| Lakes | 6 | 0.8 | 23 | 1.9 |
| MidCentral | 13 | 1 | 52 | 2.7 |
| Nelson-Marlborough | 12 | 1 | 68 | 3.8 |
| Northland | 26 | 2 | 114 | 6.0 |
| South Canterbury | 10 | 1.7 | 21 | 3.3 |
| Southern | 38 | 1.5 | 152 | 4.3 |
| Tairawhiti | 12 | 2.6 | 2 | 0.3 |
| Taranaki | 13 | 1.3 | 24 | 1.9 |
| Waikato | 46 | 1.9 | 85 | 2 |
| Wairarapa | 16 | 3.7 | 8 | 1.7 |
| Waitemata | 81 | 8.4 | 242 | 4.3 |
| West Coast | 9 | 2 | 38 | 10.6 |
| Whanganui | 9 | 1.2 | 6 | 0.8 |

**Appendix Table 7.** Legionellosis case fatality risk (CFR) by age group, sex, ethnicity and Legionella spp. New Zealand, 2000–2009 and 2010–2020

| | **2000–2009 (N = 643)** | | | **2010–2020 (N = 1985)** | | | **Risk ratio of CFR in 2010–20 to 2000–09** |
|---|---|---|---|---|---|---|---|
| **Time period** | **No. deaths** | **No. cases** | **Case-fatality risk (%)** | **No. deaths** | **No. cases** | **Case-fatality risk (%)** | **(95%CI)** |
| **Age group** | | | | | | | |
| Total all ages | 26 | 643 | 4.0 | 35 | 1985 | 1.8 | 0.4 (0.3–0.7) |
| 0–39 | 1 | 46 | 2.2 | - | 99 | - | - |
| 40–59 | 5 | 219 | 2.3 | 5 | 619 | 0.8 | 0.4 (0.1–1.2) |
| ≥60 | 20 | 378 | 5.3 | 30 | 1267 | 2.4 | 0.5 (0.3–0.8) |
| **Sex** | | | | | | | |
| Male | 18 | 383 | 4.7 | 25 | 1265 | 2.0 | 0.4 (0.2–0.8) |
| Female | 8 | 254 | 3.1 | 10 | 720 | 1.4 | 0.4 (0.2–1.1) |
| Unknown | - | 6 | - | - | 0 | - | - |
| **Ethnicity** | | | | | | | |
| European | 24 | 517 | 4.6 | 31 | 1620 | 1.9 | 0.4 (0.2–0.7) |
| Māori | - | 27 | - | - | 149 | - | - |
| Pacific Peoples | - | 13 | - | 3 | 72 | 4.2 | - |
| Other | 1 | 17 | 5.9 | 1 | 93 | 1.1 | 0.2 (0.0–3.1) |
| Unknown | 1 | 69 | 1.5 | - | 51 | - | - |
| ***Legionella* spp. (all ages)** | | | | | | | |
| *L. pneumophila* | 8 | 256 | 3.1 | 14 | 527 | 2.7 | 0.9 (0.4–2.1) |
| *L. longbeachae* | 7 | 161 | 4.3 | 11 | 1062 | 1.0 | 0.2 (0.09–0.6) |
| Other *Legionella* spp. | 2 | 87 | 2.3 | 1 | 251 | 0.4 | 0.2 (0.0–1.9) |
| Unknown | 9 | 139 | 6.5 | 9 | 145 | 6.2 | 1.0 (0.4–2.5) |
